# Systemic AL amyloidosis: current approach and future direction

**DOI:** 10.18632/oncotarget.28415

**Published:** 2023-04-26

**Authors:** Maroun Bou Zerdan, Lewis Nasr, Farhan Khalid, Sabine Allam, Youssef Bouferraa, Saba Batool, Muhammad Tayyeb, Shubham Adroja, Mahinbanu Mammadii, Faiz Anwer, Shahzad Raza, Chakra P. Chaulagain

**Affiliations:** ^1^Department of Internal Medicine, SUNY Upstate Medical University, Syracuse, NY 13210, USA; ^2^University of Texas MD Anderson Cancer Center, Houston, TX 77030, USA; ^3^Department of Internal Medicine, Monmouth Medical Center, Long Branch, NJ 07740, USA; ^4^Department of Medicine and Medical Sciences, University of Balamand, Balamand, Lebanon; ^5^Department of Internal Medicine, Cleveland Clinic, Cleveland, OH 44195, USA; ^6^Department of Internal Medicine, UnityPoint Methodist, Peoria, IL 61636, USA; ^7^Department of Internal Medicine, Monmouth Medical Center, Long Branch, NJ 07740, USA; ^8^Hematology and Medical Oncology, Houston Methodist Cancer Center, Houston, TX 77094, USA; ^9^Department of Hematology-Oncology, Cleveland Clinic, Cleveland, OH 44195, USA; ^10^Department of Hematology-Oncology, Myeloma and Amyloidosis Program, Maroone Cancer Center, Cleveland Clinic Florida, Weston, FL 33331, USA

**Keywords:** amyloidosis, management

## Abstract

Systemic Light chain (AL) amyloidosis is a monoclonal plasma cell proliferative disorder characterized by deposition of amyloidogenic monoclonal light chain fragments causing organ dysfunction. It is a fatal disease and if not diagnosed and treated early can lead to organ failure and potentially death. The renal system along with the cardiovascular system are the most common organs involved but other organs such as gut and liver can be involved as well. The initial evaluation of patients requires confirming the diagnosis with tissue biopsy and staining with Congo red followed by confirmatory typing with mass spectrometry of the Congo red positive tissue. Then establishing the extent of the organs involvement by various staging and biomarkers testing. The treatment options and the tolerability of therapy depend on the disease staging, frailty, and co-morbidities. The autologous hematopoietic cell transplantation (HCT) after high dose melphalan therapy is an effective strategy which is usually done after initial bortezomib induction therapy. Unfortunately, most systemic AL amyloidosis patients are not candidate for HCT due to frailty, old age, multi-organ involvement, renal and heart failure at the time of diagnosis. While it is widely accepted that the patients need to be treated until they achieve complete hematologic response, the maintenance therapy after HCT is not well established in AL amyloidosis. In this review, we report the literature on the latest treatment updates of AL amyloidosis and the ongoing clinical trials highlighting the future treatments.

## INTRODUCTION

Light-chain (AL) amyloidosis, also known as primary amyloidosis, is the most common and severe form of systemic amyloidosis with an estimated incidence of 10 cases per million persons a year [1–3]. It is a clonal plasma cell dyscrasia often arising in the setting of a pre-malignant monoclonal plasma cell proliferative disorder such as monoclonal gammopathy of undetermined significance or smoldering myeloma. On diagnosis, around 10% of patients with AL amyloidosis will also meet the diagnostic criteria of multiple myeloma and are excluded from AL amyloidosis and multiple myeloma clinical trials [4].

Systemic AL amyloidosis is characterized by extracellular deposition of fibrils formed by kappa (κ), lambda (λ) light chains, or their fragments [3]. Localized AL amyloidosis does not usually need systemic chemoimmunotherapy and therefore is not discussed in this paper. Amyloids can accumulate in various tissues, leading to organ dysfunction and failure. Thus, the heterogeneous and insidious organ-dependent clinical presentation makes it difficult to recognize, with common misdiagnosis and delayed diagnosis of about 6 months to 1 year after symptoms onset [4]. Nearly 70% of patients have multiple organ involvement by the time of diagnosis [3]. Notably, the kidneys and heart are the most frequently affected organs, followed by the liver, the gastrointestinal tract, and the autonomic nervous system [3, 5]. Nevertheless, myocardial infiltration leading to heart failure carries the worst outcomes and urges to the need of early diagnosis [6]. Unexplained diastolic heart failure especially with a restrictive pattern should raise suspicion for amyloidosis [6, 7]. Tissue biopsy is the gold standard requirement for the diagnosis of AL amyloidosis while biomarkers and imaging studies can be suggestive [6]. Congo Red and Thioflavin S are the two major histological stains used to detect any form of amyloid. Upon Congo Red staining of the tissue specimen, the sample should demonstrate green birefringence fibrils on a polarized microscope. Additional typing can be done using immunochemistry or mass spectrometry [4]. The biopsy is usually preceded by an extensive diagnostic workup including but not limited to: serum free light chain assay, serum and urine protein electrophoresis with immunofixation, a complete blood count, liver and renal function tests, 24-hour urine protein and quantitative immunoglobulin levels [5, 6]. There is a strong relationship between AL amyloidosis and monoclonal plasma cell proliferative disorders, particularly multiple myeloma. In fact, up to 15–20% of patients with multiple myeloma will develop AL amyloidosis. In addition, up to 10% of patients with AL amyloidosis may have an underlying monoclonal plasma cell disorder [8].

The relationship between AL amyloidosis and MGUS is less clear, but some studies suggest that the risk of developing AL amyloidosis may be increased in patients with MGUS. It is important for patients with these conditions to undergo regular monitoring and evaluation for signs of AL amyloidosis, as early diagnosis and treatment can improve outcomes.

In this manuscript, we discuss the general approach towards treating patients with amyloidosis and dive into the future perspectives in this multi-systemic disease.

## CARDIAC INVOLVEMENT

Myocardial infiltration refers to the infiltration of abnormal substances, such as amyloid proteins or cancer cells, into the heart muscle. Early diagnosis of myocardial infiltration is important because it is associated with poor outcomes, including heart failure, arrhythmias, and sudden cardiac death.

One of the most common causes of myocardial infiltration is cardiac amyloidosis, a disease in which abnormal proteins called amyloid fibrils deposit in the heart muscle. Amyloidosis is often underdiagnosed or misdiagnosed due to its non-specific symptoms and rarity, but early diagnosis is critical for improving outcomes [9–11].

One of the challenges with diagnosing cardiac amyloidosis is that its symptoms are like those of other cardiac diseases, such as hypertrophic cardiomyopathy or heart failure. Therefore, a high index of suspicion is required, especially in patients with unexplained heart failure or arrhythmias.

Early diagnosis of cardiac amyloidosis is important because the disease is often progressive and irreversible. The prognosis for patients with untreated cardiac amyloidosis is poor, with a median survival of less than 2 years. However, with early diagnosis and treatment, outcomes can be improved, and some patients may even experience a reversal of cardiac damage.

Treatment options for cardiac amyloidosis include medications that target the underlying cause of the disease, such as chemotherapy for AL amyloidosis, and supportive measures to manage symptoms and prevent complications [9–11].

In summary, early diagnosis of myocardial infiltration, particularly cardiac amyloidosis, is crucial for improving outcomes and preventing the progression of the disease. It requires a high index of suspicion, and patients with unexplained heart failure or arrhythmias should be evaluated for the possibility of cardiac amyloidosis [9–11].

## STAGING

In systemic AL amyloidosis, the extent of cardiac involvement determines prognosis and treatment response. Serum cardiac biomarkers NT-pro-BNP and troponin T reflect the extent of cardiac involvement and guide the initial cardiac staging and treatment strategy [6]. The Mayo 2004 staging system uses troponin T (TnT), N-terminal pro-B-type natriuretic peptides (NT-proBNP) to categorize AL amyloidosis patients according to risk status and predict overall survival (OS) (Table 1). The BNP is the active form of the hormone, NT-proBNP is passively cleared from the blood and has a greater half-life making it less subject to hemodynamic variations [12]. BNP can also be used when testing for NT-proBNP is not available. Another advantage of NT-proBNP is that it has a higher diagnostic accuracy for detecting cardiac involvement in patients with AL amyloidosis than BNP. Studies have shown that NT-proBNP levels are more strongly correlated with echocardiographic measures of cardiac function, such as left ventricular wall thickness and ejection fraction, than BNP levels in patients with AL amyloidosis. Finally, NT-proBNP is also easier to measure than BNP, as it is not affected using medications that interfere with BNP assays, such as neprilysin inhibitors [13]. The European 2016 modification of the Mayo 2004 staging system utilizes similar cut-off levels for the aforementioned cardiac markers but introduces a new stage by splitting stage III into stage IIIA and IIIB where NT-proBNP is exceedingly high [14]. The Mayo 2012 staging system incorporates the absolute difference between involved and uninvolved free light chains (dFLC) into the prognostic criteria as a measure of the hematologic burden of the disease [14].

**Table 1:** Staging system

Staging system	Risk factors			Stage		Median survival (1) (Months or % at 3 year)	Hazard ratio for death
	Cardiac markers threshold		Hematological markers threshold				
	TnT (μg/L)	NT-proBNP (ng/L)	dFLC (mg/L)				
**Mayo 2004**	≥0.035	≥332	–	I	No risk factors	27.2 mo	(Range)
				II	1 risk factor	11.1 mo	2.5 (1.9–3.5)
				III	All risk factors apply	4.1 mo	6.7 (5.0–9.1)
**European 2016** **modification of** **Mayo 2004**	≥0.035	≥332	–	I	No risk factors	100%	(Range)
				II	At least one risk factor	52%	2.5 (1.9–3.5)
				IIIA	All risk factors apply	55%	4.9 (3.6–6.8)
		≥8500		IIIB	All risk factors apply	19%	11.1 (8.1–15.4)
**Mayo 2012**	≥0.025	≥1800	≥180	I	No risk factors	94.1 mo	(Range)
				II	1 risk factor/3	40.3 mo	1.7 (1.2–2.3)
				III	2 risk factors/3	14 mo	4.1 (3.1–5.5)
				IV	All risk factors apply	5.8 mo	6.3 (4.8–8.3)

## INDICATIONS FOR SYSTEMIC THERAPY

The goal of current therapy in AL amyloidosis is reducing the amyloidogenic immunoglobulin free light chain levels as rapidly as possible by using the most effective anti-plasma cell therapy along with supportive care by management of the secondary organ dysfunction or organ failure. In general, at the time of diagnosis, all systemic AL amyloidosis requires systemic treatment. However, there are a few exceptions, like localized AL Amyloidosis. Localized AL Amyloidosis is generally present in a single organ, such as the lungs, part of the bowel, upper airways, or skin along with the absence of the monoclonal protein in the plasma and absence of clonal plasma cells in the bone marrow [15–17]. Localized AL amyloidosis is not treated by systemic therapy since the long-term prognosis is favorable, and not complicated with systemic AL amyloidosis and is generally managed by local resection [18]. While a monoclonal gammopathy of unknown significance (MGUS) may develop to systemic amyloidosis or multiple myeloma with an average rate of transformation of 1% per year [19, 20], finding of AL amyloidosis in bone marrow is not an indication for systemic treatment in the absence of the organ involvement. This approach is also true in smoldering multiple myeloma with a higher risk of transformation to multiple myeloma of 10% per year [20]. In terms of making decision regarding systemic treatment in AL amyloidosis, the provider needs to account the patient’s overall performance status, level of organ dysfunction, and whether the patient is a candidate for HCT [21].

After confirming the diagnosis of AL amyloidosis, all patients will undergo evaluation to assess their eligibility for autologous hematopoietic cell transplantation (HCT). The transplant eligibility will determine the next steps of treatment which are different between transplant eligible and transplant ineligible patients. It is estimated that 20% of the patients with newly diagnosed AL amyloidosis are candidates for HCT at the time of diagnosis and 80% of patients are not eligible for this effective therapy. The eligibility for HCT is increasing due to the availability of effective up-front induction therapy based on daratumumab and bortezomib as such therapy can convert an otherwise HCT ineligible patient to HCT eligible if the patient achieves rapid hematologic and organ response [22].

Eligibility criteria for HCT usually vary between centers depending on specific policies and local experience. While HCT is commonly offered for people below the age of 70, carefully selected patients above the age of 70 years can have good outcomes with HCT [23]. In a retrospective study at Mayo Clinic Rochester, an overall response rate of 75% and a complete response rate of 25% was noted after HCT in patients with AL amyloidosis above the 70 over 15 years [23]. The post-HCT hospitalization rates and 100-day mortality were comparable to the reported rates in patients younger than 70 years old, indicating that HCT can be an efficacious and safe therapeutic intervention in AL amyloidosis patients above the age of 70 [23]. As such, there is no strict age cutoff and decisions are made on a case-by-case basis based on the “physiologic” rather than the “chronologic” age of patients. Patients older than 70 years of age are usually discussed in multidisciplinary meetings and preferably referred to extensively to high volume HCT experienced centers [24]. The eligibility criteria for HCT in amyloidosis patients have been summarized in Table 2.

**Table 2:** Eligibility criteria for HCT in AL systemic amyloidosis patients

Eligibility criteria
• Age >18 years old, with a “physiologic age” <70
• Confirmed tissue diagnosis of AL amyloidosis with appropriate typing
• Evidence of clonal plasma cell dyscrasia
• At least 1 major vital organ involvement (solitary amyloid deposition in the bone marrow or soft tissue involvement is not included)
• No more than 2 vital organs significantly involved (Heart, autonomic nervous system, kidney, liver)
• ECOG performance status of ≤ 2 (exceptions are considered if the peripheral neuropathy is contributing to the advanced ECOG status)
• A room air blood oxygen saturation ≥95%, with a DLCO >50%
• Supine systolic blood pressure ≥90 mmHg
• Absence of orthostatic hypotension that is refractory to medical therapy
• Left ventricular ejection fraction ≥40%, with a NYHA class <III
• Absence of decompensated heart failure
• Absence of symptomatic or medically refractory atrial or ventricular arrythmias
• Absence of symptomatic or medically refractory pleural effusions
• Absence of significant gastrointestinal tract involvement with active or increased risk of bleeding
• Conjugated bilirubin <2 mg/dL
• NTproBNP <5000 pg/mL
• Troponin I <0.1 ng/mL, Troponin T <60 ng/ML and hs-Troponin <75 ng/mL
• eGFR >30 mL/min/m^2*^
• Absence of severe factor X deficiency (defined as factor X levels <25%)^**^

^*^Patients with eGFR ≤30 mL/min/m^2^ and not yet on dialysis at the time of the evaluation are at increased risk of worsening kidney function with HCT (20). Patients with end stage renal disease with a known stable dialysis schedule should not be excluded from getting HCT if they meet all the other eligibility criteria. ^**^Patients with severe deficiency have a transplant-related mortality rate that approaches 50%. Splenectomy however can be performed in those patients to increase their factor X levels prior to HCT.

These criteria have been established after multiple retrospective and small prospective studies have shown worse adverse outcomes of HCT in patients with AL amyloidosis with extensive organ involvement [25–27]. For example, in a series of 21 patients, patients with less than 2 amyloidosis manifestations (criteria included in the study included eGFR ≤30 mL/min/m^2^, nephrotic syndrome, neuropathy, hepatomegaly with alkaline phosphatase concertation >200 IU/L, heart failure) had significantly higher overall and event-free survival than those with more than 2 manifestations. This was mostly attributed to the high incidence of toxicity and death in the latter group, reaching up to 75% [27].

### Bortezomib based regimen

Historically, a preferred management for transplant eligible and ineligible newly diagnosed patients was debatable but often included cyclophosphamide, bortezomib, dexamethasone and cyclophosphamide (CyBorD). The results of phase III randomized ANDROMEDA study has shifted that paradigm: In patients with newly diagnosed AL amyloidosis, the addition of subcutaneous anti-CD39 monoclonal antibody daratumumab to CyBorD (Dara-CyborD) was associated with higher frequencies of hematologic complete response and survival free from major organ deterioration or hematologic progression [28]. Based on these data, Dara-CyborD is approved as the new standard of care for induction therapy in newly diagnosed AL amyloidosis patients. In a median follow-up of 11.4 months in this study with *N* = 388 newly diagnosed AL amyloidosis patients, the hematologic complete response was significantly higher in the daratumumab group than in the control group (53.3% vs. 18.1%), and at 6 months, more cardiac and renal responses occurred in the daratumumab group than in the control group (41.5% vs. 22.2% and 53.0% vs. 23.9%, respectively). The addition of daratumuamb to CyBorD was tolerable without any significant added side effects.

If daratumumab is not available or not affordable (such as in resource poor countries) bortezomib based induction should be considered. In a prospective observational study, Manwani et al. reported a total of 915 patients with newly diagnosed AL amyloidosis who were treated with bortezomib based therapy. Responses observed were hematologic complete response (CR) seen in 25% patients, overall response rate (ORR) was 65% and median overall survival (OS) was 72 months [29].

The preference of bortezomib with melphalan and dexamethasone is based on (EMN-03) trial that compared addition of Bortezomib to melphalan and dexamethasone (BMDex) to Melphalan and dexamethasone-based regimen (MDex). BMDex resulted in significant improvements in overall response (OR) (81% vs. 57%) and overall survival (OS). This was also the first controlled study demonstrating improved survival outcome in AL amyloidosis [30].

A meta-analysis involving three randomized controlled trials (RCTs) and thirteen observational controlled trials (OCTs) comparing seven treatment regimens: High dose melphalan followed by autologous stem cell transplant (HDM/HSCT), Melphalan + dexamethasone (MDex), Bortezomib + melphalan + dexamethasone (BMDex), Bortezomib + dexamethasone (BDex), Thalidomide + cyclophosphamide + dexamethasone (CTD), Bortezomib + Cyclophosphamide + dexamethasone (CyBorD), cyclophosphamide + lenalidomide + dexamethasone (CLD) showed BMDex ranking high in terms of hematologic response and complete response (CR), CTD ranking high in terms of renal response and BDex was best treatment for cardiac response [31].

### Melphalan based regimen

High dose melphalan and autologous stem cell transplant (HCT) is an effective treatment option with a high hematologic response rate and a prolonged overall survival rate with durable remission [32]. However, for transplant ineligible patients, oral melphalan and prednisone was used in the past which is rarely used in the USA in current hematology practice. Palladini et al. reported a study involving 259 subjects where 119 were given melphalan and a full dose dexamethasone and the rest of subjects received Melphalan with attenuated dose dexamethasone due to cardiac involvement. The study showed improved hematologic response and complete response in Melphalan and dexamethasone group with minimal toxicity compared to attenuated steroid dosage group. A bortezomib regimen was considered a rescue treatment for relapsed/refractory disease [33]. The melphalan and dexamethasone can still be considered an option for resource poor setting when the patient is not a candidate for HCT and a daratumumab or bortezomib based regimen is not available.

The efficacy of a lenalidomide treatment in Myeloma led to the search for efficacy and utility of lenalidomide in Amyloidosis. A prospective phase II trial of lenalidomide, melphalan and dexamethasone involving sixteen subjects showed that hematologic response was achieved by 50%, with partial response (PR) and complete response (CR) of 47% and 7% respectively. All responses were evident within 3 months of starting protocol treatment. Of 16 patients initially enrolled, 14 completed at least 3 cycles of treatment and were evaluated for response [34]. Immunomodulatory drugs (IMiDs) are not used frontline in the treatment of AL amyloidosis due to tolerability issues particularly with lenalidomide (can increase creatinine and cardiac biomarkers).

## FUTURE PERSPECTIVES IN SYSTEMIC AL AMYLOIDOSIS

CAEL-101 is a monoclonal antibody that acts by binding to amyloid light chain fibrils promoting the clearance of amyloid light chain fibrils from affected tissues. It has been tested in a recently conducted open-label Phase Ia/Ib clinical trial to determine the tolerability and potential amyloid depleting effect of monoclonal antibody (mAb) CAEL-101 [35]. From September 2014 to April 2017, 27 patients were enrolled, and eligible patients included adults ≥21 years with confirmed histopathologic diagnosis of AL Amyloidosis who received prior systemic therapy and had an Eastern Cooperative Oncology Group performance status of ≤3. Patients were excluded for seriously limited vital organs functions (intraventricular septum thickness >25 mm or Ejection Fraction <40%), renal (creatinine clearance ≤30 mL/min), or hepatic function (alkaline phosphatase ≥3.0 times the upper limit of normal and total bilirubin ≥3.0 mg/dL), uncontrolled infection, or significant comorbidity [33]. Patients with systemic AL Amyloidosis and persistent organ disease were treated in 1a (*n* = 8) and 1b (*n* = 19). In phase 1a, patients received mAb CAEL-101 as a single intravenous infusion with escalating dose levels from 0.5 mg/m^2^ to 500 mg/m^2^ to establish the maximum tolerated dose (MTD). In phase 1b, the antibody was administered as a graded series of 4 weekly infusions [33]. In the trial, 19 patients were male and 8 were female patients. In the overall population, the median age was 66 years (range 34–79). Among the total number of patients, 20 were White Americans, 1 patient belonged to the African American population, and 6 belonged to ethnicities other than White and African Americans. The median number of organs involved was 2 (range 1–4) [33]. Most patients had cardiac (59.3%, 16 of 27) and renal involvement (48.1%, 13 of 27). The median number of prior anti-plasma cell regimens was 2 (range 1–10). The median time since exposure to chemotherapy was 2.6 and 7.4 months (range 0–15.5 months) in phase 1a and 1b, respectively. There were no Dose Limited Toxicities (DLTs) noted up to 500 mg/m^2^ in Phase 1a or 1b. The most common treatment-related adverse events in phase 1a included grade 1 to 2 nausea, diarrhea, rash, pruritus, and hyperuricemia [33]. In phase 1b, diarrhea, rash, and aspartate transaminase elevation were the most common adverse events. 1 patient (5.3%) experienced grade 3 pericardial effusion due to advanced cardiac amyloidosis which was observed without intervention. Out of 27 patients, 3 patients had no measurable disease. Therefore, 24 patients were evaluated for response. Of the cardiac-evaluable patients, 67% showed organ response, 33% had stable disease, and no patients met the criteria for cardiac progression. In the response analysis for renal evaluable patients, 20% showed organ response, 60% patients had stable disease and 20% patients met criteria for disease progression. Three patients with involvement of other organs had clinical improvement while on treatment. In both phase 1a and 1b, organ responses were noted in evaluable patients receiving 5 mg/m^2^ of the antibody [33]. There were no organ responses at lower doses. The overall organ response rate was 63% in those receiving at least 1 dose of mAb CAEL-101. The median time to response was 3 weeks after the first infusion of mAb CAEL-101. Valent et al. reported the 1-year results from a phase 2 study to determine the safety and tolerability of treating patients with Light-Chain Amyloidosis with CAEL-101. A total of 25 patients with Mayo Stage I (8%), II (76%), and IIIa (16%) with AL Amyloidosis were treated with CAEL-101 for 1 year. The mean age of the enrolled patients was 65 years and 18 (72%) were males. Patients presented with cardiac involvement (*n* = 22), renal involvement (*n* = 9), and a prior plasma cell dyscrasia therapy (*n* = 20). The cardiac and renal response was assessed by a ≥30% decrease in N-terminal pro-brain natriuretic peptide (NT-proBNP) and proteinuria respectively. After 1 year of CAEL-101 treatment of the 22 cardiac evaluable patients (baseline NT-proBNP ≥332 pg/mL), 10 (46%) patients experienced ≥30% NT-proBNP decrease from baseline, 4 (18%) were stable (±30% change from baseline), and 3 (14%) showed disease progression (≥30% NT-proBNP increase from baseline); data for 5 patients were missing. Of the 9 renal evaluable patients from a single site as reported by the investigator, 8 showed ≥30% decrease from baseline in proteinuria. All 25 (100%) patients experienced TEAEs and 6 (24%) experienced possibly treatment related TEAEs. 15 (60%) patients experienced TEAEs of Grade ≥3 severity and 13 (52%) experienced ≥1 serious adverse event (SAE). The most common TEAEs were nausea (*n* = 10), constipation (*n* = 9), fatigue (*n* = 9), anemia, insomnia, or diarrhea (*n* = 9 each), and dizziness, cough, or rash (*n* = 7 each). Various Clinical trials are going for the effective treatment of AL Amyloidosis. Birtamimab is another monoclonal antibody that neutralizes circulating soluble and depletes deposited insoluble amyloid by promoting phagocytic clearance. In 2018, the VITAL Study; A Phase 3, Randomized, Multicenter, Double-Blind study evaluated the Efficacy and Safety Study of NEOD001 (Birtamimab) Plus Standard of Care Versus Placebo Plus Standard of Care in Subjects with Light Chain (AL) Amyloidosis. Gertz et al. reported the results of sensitivity analyses of All-cause mortality (ACM) in a subgroup of patients with Mayo 2012 Stage IV AL Amyloidosis. Of the 260 patients enrolled in the VITAL study, 77 subjects were characterized as Mayo 2012 Stage IV at baseline. Out of 77 patients, 38 were randomized to Birtamimab + Standard of Care (SOC) and 39 to placebo + SOC, and the median age was 64 years. Baseline demographics and clinical characteristics were balanced between the two treatment groups. After adjustment for key baseline demographic, clinical, and laboratory variables, a pronounced survival benefit was found (HR = 0.413, 95% CI 0.191–0.895 *p* = 0.025). The phase-3, randomized, multicenter, double-blind, placebo-controlled AFFIRM-AL study is designed to confirm the VITAL study results in Mayo Stage IV AL Amyloidosis patients. The primary objective of this study is to evaluate the efficacy of Birtamimab by assessing time to all-cause mortality. Approximately 150 newly diagnosed Mayo Stage IV patients with AL amyloidosis are expected to be enrolled and randomized in a 2:1 ratio to Birtamimab or placebo. Table 3 mentions a few of the major clinical trials currently addressing treatment regimens in Amyloidosis.

**Table 3:** Amyloidosis treatment clinical trials (https://clinicaltrials.gov)

Clinical trial name and NCT	Intervention	Patient population	Primary outcome
Daratumumab, Ixazomib, and Dexamethasone in AL Amyloidosis NCT03283917 Phase I	Biological Agent: Daratumumab Drug: Dexamethasone & Ixazomib	N/A	Dose Limiting Toxicity Rate Recommended Phase 2 dose of Daratumumab, Ixazomib, and Dexamethasone
Bortezomib/Dexamethasone, followed by Autologous Stem Cell Transplantation and Maintenance Bortezomib/Dexamethasone NCT01383759	Drug: Bortezomib/Dexamethasone (BD), Followed by Autologous STC and Maintenance Bortezomib/ Dexamethasone	White-84%, Asian-5.3%, African American-5.3%, Unknown-5.3%	Percentage of participants Experiencing PFS at 12 months Participants evaluated for Toxicity
Lenalidomide, Dexamethasone, and Elotuzumab with or without Cyclophosphamide in Treating Patients with Relapsed Primary Amyloidosis NCT03252600 (Phase II)	Experimental: Arm I (lenalidomide, dexamethasone, Elotuzumab) Experimental: Arm II (lenalidomide, dexamethasone, elotuzumab, cyclophosphamide)	N/A	Major Hematologic Response (≥ very good partial response), or better
Isatuximab as Upfront Therapy for the Treatment of High-Risk AL Amyloidosis NCT04754945 (Phase I)	Drugs: Bortezomib, Cyclophosphamide, Dexamethasone Biological: Isatuximab	N/A	Event-free proportion [Time Frame: 3 months]
Trial of Venetoclax (ABT-199) and Dexamethasone for Relapsed or Refractory Systemic AL Amyloidosis NCT03000660 (Phase I)	Drug: Venatoclax Drug: Dexamethasone	N/A	Participants with treatment related adverse events using NCI CTCAE version 4.03. (Time frame up to 8 months after beginning of the study)
A Study to Evaluate the Efficacy and Safety of CAEL-101 in Patients with Mayo Stage IIIa AL Amyloidosis NCT04512235 (Phase III)	Experimental Arm: CAEL-101 + cyclophosphamide, bortezomib, and dexamethasone (CyBorD) regimen Placebo arm: Placebo + cyclophosphamide, bortezomib, and dexamethasone (CyBorD) regimen	N/A	Time from the date of randomization to date of death or end of study Number of patients with treatment emergent adverse events as assessed by CTCAE v5.0
A Study to Evaluate the Efficacy and Safety of CAEL-101 in Patients with Mayo Stage IIIb AL Amyloidosis NCT04504825 (Phase III)	Experimental Arm: CAEL-101 + cyclophosphamide, bortezomib, and dexamethasone (CyBorD) regimen Placebo arm: Placebo + cyclophosphamide, bortezomib, and dexamethasone (CyBorD) regimen	N/A	Time from the date of randomization to date of death or end of study Number of patients with treatment emergent adverse events as assessed by CTCAE v5.0
Selinexor for Treatment of Light Chain Amyloidosis with Relapsed/ Refractory Disease (STARR) NCT04984330 (Early Phase I)	Selinexor and Dexamethasone	N/A	Compare number of dose limiting toxicity (DLT) occurrence to measure safety and toxicity (Time Frame: approx 12 months)
Renal AL Amyloid Involvement and NEOD001 (RAIN) NCT03168906 (Phase 2)	Experimental Arm: NEOD001 Comparator arm: Placebo 12 participants (6 in each)	White 10 (83.3%) African American 2 (16.7%)	Confirmed Renal Response After Treatment with NEOD001 (Time Frame: Baseline to 13 Months)
S1702 Isatuximab in Treating Patients with Relapsed or Refractory Primary Amyloidosis NCT03499808 (Phase II)	Isatuximab	N/A	Overall confirmed hematologic response rate (Time Frame: Up to 4 years)
Venetoclax, Daratumumab, and Dexamethasone for Systemic Light-Chain Amyloidosis with Translocation (11;14) (ALTITUDE) NCT05486481 (Phase I & II)	Venetoclax, Daratumumab, and Dexamethasone	N/A	Proportion of participants with reported dose limiting toxicities (Phase 1a) [Time Frame: Up to 1 cycle (1 cycle is equal to 28 days)] Maximum tolerated dose (MTD) (Phase 1a) [Time Frame: Up to 1 cycle (1 cycle is equal to 28 days)] Recommended phase 2 dose (RP2D) (Phase1a) [Time Frame: Up to 1 cycle (1 cycle is equal to 28 days)] Percentage of participants with treatment-emergent adverse events attributable to study treatment (Phase 1b/2) [Time Frame: Up to 2 years] Proportion of participants who achieve a complete hematologic response (CHR) (Phase 2) [Time Frame: Up to 2 years]
Study of Oral Ixazomib in Adult Participants with Relapsed or Refractory Light Chain Amyloidosis NCT01318902 (Phase I)	Ixazomib & Dexamethasone 4 Experimental arms 2 experimental arms with Dose escalation and 2 arms with dose expansion	White-23/27 (85.2%) African American-1 (3.2%) Not reported 1 (3.2%) Other-1 (3.2%)	Treatment Emergent Adverse Event (TEAE) and Serious Adverse Event (SAE) Number of Participants with Clinically Significant Abnormal Laboratory Values Peripheral Neuropathy Reported as a TEAE Maximum Tolerated Dose (MTD) of Ixazomib Recommended Phase 2 Dose (RP2D) of Ixazomib
Venetoclax, MLN9708 (Ixazomib Citrate) and Dexamethasone for the Treatment of Relapsed or Refractory Light Chain Amyloidosis NCT04847453	Drug: Venetoclax, Dexamethasone, and Ixazomib	N/A	Incidence of adverse events Maximum Tolerated Dose Recommended Phase 2 dose

## CONCLUSION

Light chain amyloidosis is a clonal plasma cell dyscrasia characterized by the extracellular depositions of fragments of immunoglobulin light or heavy chain in the tissues leading to organ dysfunction and failure. Clinical features depend on organs involved but the kidneys and heart are most frequently affected organs, followed by gastrointestinal tract and peripheral nervous system. A tissue biopsy stained with Congo red demonstrating amyloid deposits clinches the diagnosis followed by a confirmatory typing by mass spectroscopy. AL amyloidosis is a fatal disease and systemic therapy is required to prevent deposition of amyloid in other organs and prevent progressive organ failure. Current first line induction therapy is daratumumab combined with bortezomib, cyclophosphamide and dexamethasone (Dara-CyBorD). Autologous stem cell transplantation after high dose melphalan therapy (HCT) should be considered for eligible patients. Novel and evolving therapy include BCL-2 inhibitor venetoclax in AL patients with t(11;14). Though not FDA approved yet, venetoclax is currently being used off the label for relapsed/refractory AL amyloidosis patients with good efficacy and excellent tolerability and safety. Venetoclax is being investigated in relapsed refractory setting as well as up front in combination with daratumumab. Selinexor is another oral therapy under clinical investigation in AL amyloidosis. Finally, the clinical trials of new treatments against deposited amyloid fibrils include monoclonal antibodies such as CAEL 101 or Birtamimab which if found effective and approved will revolutionize the treatment against AL amyloidosis.

## Abbreviations

AL: Light chain
BD: Bortezomib/Dexamethasone
BMDex: Bortezomib, melphalan and dexamethasone
CHR: complete hematologic response
CKDEPI: Chronic Kidney Disease Epidemiology Collaboration
CR: complete response
Dara-CyborD: Daratumumab, Cyclophosphamide
DLCO: diffusion capacity of the lungs for carbon monoxide
dFLC: uninvolved free light chains
DLTs: Dose Limited Toxicities
eGFR: glomerular filtration rate
HDM/HSCT: High dose melphalan and autologous stem cell transplant
HCT: hematopoietic cell transplantation
κ: kappa
λ: lambda
MDex: Melphalan, dexamethasone based regimen
MTD: maximum tolerated dose
NT-proBNP: N-terminal pro-B-type natriuretic peptides
ORR: overall response rate
OS: overall survival
RP2D: Recommended phase 2 dose
RCTs: randomized controlled trials
SAE: Serious Adverse Event
TEAE: Treatment Emergent Adverse Event
TnT: troponin T

## References

[1] Gertz MA , Dispenzieri A . Systemic Amyloidosis Recognition, Prognosis, and Therapy: A Systematic Review. JAMA. 2020; 324:79–89. 10.1001/jama.2020.5493. 32633805

[2] Muchtar E , Dispenzieri A , Gertz MA , Kumar SK , Buadi FK , Leung N , Lacy MQ , Dingli D , Ailawadhi S , Bergsagel PL , Fonseca R , Hayman SR , Kapoor P , et al. Treatment of AL Amyloidosis: Mayo Stratification of Myeloma and Risk-Adapted Therapy (mSMART) Consensus Statement 2020 Update. Mayo Clin Proc. 2021; 96:1546–77. 10.1016/j.mayocp.2021.03.012. 34088417

[3] Ryšavá R . AL amyloidosis: advances in diagnostics and treatment. Nephrol Dial Transplant. 2019; 34:1460–66. 10.1093/ndt/gfy291. 30299492

[4] Oubari S , Naser E , Papathanasiou M , Luedike P , Hagenacker T , Thimm A , Rischpler C , Kessler L , Kimmich C , Hegenbart U , Schönland S , Rassaf T , Reinhardt HC , et al. Impact of time to diagnosis on Mayo stages, treatment outcome, and survival in patients with AL amyloidosis and cardiac involvement. Eur J Haematol. 2021; 107:449–57. 10.1111/ejh.13681. 34185342

[5] Gertz MA . Immunoglobulin light chain amyloidosis: 2020 update on diagnosis, prognosis, and treatment. Am J Hematol. 2020; 95:848–60. 10.1002/ajh.25819. 32267020

[6] Elsayed M , Usher S , Habib MH , Ahmed N , Ali J , Begemann M , Shabbir SA , Shune L , Al-Hilli J , Cossor F , Sperry BW , Raza S . Current Updates on the Management of AL Amyloidosis. J Hematol. 2021; 10:147–61. 10.14740/jh866. 34527111 PMC8425803

[7] Jensen CE , Byku M , Hladik GA , Jain K , Traub RE , Tuchman SA . Supportive Care and Symptom Management for Patients With Immunoglobulin Light Chain (AL) Amyloidosis. Front Oncol. 2022; 12:907584. 10.3389/fonc.2022.907584. 35814419 PMC9259942

[8] Madan S , Dispenzieri A , Lacy MQ , Buadi F , Hayman SR , Zeldenrust SR , Rajkumar SV , Gertz MA , Kumar SK . Clinical features and treatment response of light chain (AL) amyloidosis diagnosed in patients with previous diagnosis of multiple myeloma. Mayo Clin Proc. 2010; 85:232–38. 10.4065/mcp.2009.0547. 20194151 PMC2843113

[9] Ash S , Shorer E , Ramgobin D , Vo M , Gibbons J , Golamari R , Jain R , Jain R . Cardiac amyloidosis-A review of current literature for the practicing physician. Clin Cardiol. 2021; 44:322–31. 10.1002/clc.23572. 33595871 PMC7943900

[10] Falk RH . Diagnosis and management of the cardiac amyloidoses. Circulation. 2005; 112:2047–60. 10.1161/CIRCULATIONAHA.104.489187. 16186440

[11] Kittleson MM , Maurer MS , Ambardekar AV , Bullock-Palmer RP , Chang PP , Eisen HJ , Nair AP , Nativi-Nicolau J , Ruberg FL , and American Heart Association Heart Failure and Transplantation Committee of the Council on Clinical Cardiology. Cardiac Amyloidosis: Evolving Diagnosis and Management: A Scientific Statement From the American Heart Association. Circulation. 2020; 142:e7–22. 10.1161/CIR.0000000000000792. 32476490

[12] Lee Chuy K , Drill E , Yang JC , Landau H , Hassoun H , Nahhas O , Chen CL , Yu AF , Steingart RM , Liu JE . Incremental Value of Global Longitudinal Strain for Predicting Survival in Patients With Advanced AL Amyloidosis. JACC CardioOncol. 2020; 2:223–31. 10.1016/j.jaccao.2020.05.012. 33117993 PMC7591133

[13] Merlini G , Lousada I , Ando Y , Dispenzieri A , Gertz MA , Grogan M , Maurer MS , Sanchorawala V , Wechalekar A , Palladini G , Comenzo RL . Rationale, application and clinical qualification for NT-proBNP as a surrogate end point in pivotal clinical trials in patients with AL amyloidosis. Leukemia. 2016; 30:1979–86. 10.1038/leu.2016.191. 27416985 PMC5056962

[14] Baker KR . Light Chain Amyloidosis: Epidemiology, Staging, and Prognostication. Methodist Debakey Cardiovasc J. 2022; 18:27–35. 10.14797/mdcvj.1070. 35414848 PMC8932379

[15] Guillet C , Steinmann S , Maul JT , Kolm I . Primary Localized Cutaneous Amyloidosis: A Retrospective Study of an Uncommon Skin Disease in the Largest Tertiary Care Center in Switzerland. Dermatology. 2022; 238:579–86. 10.1159/000518948. 34525472 PMC9153345

[16] Shaker N , Tynski Z , Hudlak M , Brown RR , Qasem S , Parwani A . Primary localized amyloidosis of the ureter with osseous metaplasia presenting as a suspicious ureteral mass. Urol Case Rep. 2022; 41:101967. 10.1016/j.eucr.2021.101967. 34950565 PMC8671856

[17] Chandra A , Khan S , Dhabal A , Banerjee G . Localized Amyloidosis of Palate. Indian Dermatol Online J. 2022; 13:425–26. 10.4103/idoj.idoj_405_21. 36226028 PMC9549550

[18] Lin XY , Pan D , Sang LX , Chang B . Primary localized gastric amyloidosis: A scoping review of the literature from clinical presentations to prognosis. World J Gastroenterol. 2021; 27:1132–48. 10.3748/wjg.v27.i12.1132. 33828390 PMC8006099

[19] Kyle RA , Larson DR , Therneau TM , Dispenzieri A , Kumar S , Cerhan JR , Rajkumar SV . Long-Term Follow-up of Monoclonal Gammopathy of Undetermined Significance. N Engl J Med. 2018; 378:241–49. 10.1056/NEJMoa1709974. 29342381 PMC5852672

[20] Fend F , Dogan A , Cook JR . Plasma cell neoplasms and related entities-evolution in diagnosis and classification. Virchows Arch. 2023; 482:163–77. 10.1007/s00428-022-03431-3. 36414803 PMC9852202

[21] Bomsztyk J , Khwaja J , Wechalekar AD . Recent guidelines for high-dose chemotherapy and autologous stem cell transplant for systemic AL amyloidosis: a practitioner’s perspective. Expert Rev Hematol. 2022; 15:781–88. 10.1080/17474086.2022.2115353. 36039749

[22] Palladini G , Milani P , Merlini G . Management of AL amyloidosis in 2020. Blood. 2020; 136:2620–27. 10.1182/blood.2020006913. 33270858

[23] Sidiqi MH , Aljama MA , Muchtar E , Buadi FK , Warsame R , Lacy MQ , Dispenzieri A , Dingli D , Leung N , Gonsalves WI , Kapoor P , Kourelis TV , Hogan WJ , et al. Autologous Stem Cell Transplant for Immunoglobulin Light Chain Amyloidosis Patients Aged 70 to 75. Biol Blood Marrow Transplant. 2018; 24:2157–59. 10.1016/j.bbmt.2018.06.017. 29933071 PMC6239955

[24] Sanchorawala V , Boccadoro M , Gertz M , Hegenbart U , Kastritis E , Landau H , Mollee P , Wechalekar A , Palladini G . Guidelines for high dose chemotherapy and stem cell transplantation for systemic AL amyloidosis: EHA-ISA working group guidelines. Amyloid. 2022; 29:1–7. 10.1080/13506129.2021.2002841. 34783272

[25] Tandon N , Muchtar E , Sidana S , Dispenzieri A , Lacy MQ , Dingli D , Buadi FK , Hayman SR , Chakraborty R , Hogan WJ , Gonsalves W , Warsame R , Kourelis TV , et al. Revisiting conditioning dose in newly diagnosed light chain amyloidosis undergoing frontline autologous stem cell transplant: impact on response and survival. Bone Marrow Transplant. 2017; 52:1126–32. 10.1038/bmt.2017.68. 28394369

[26] Goodman HJ , Gillmore JD , Lachmann HJ , Wechalekar AD , Bradwell AR , Hawkins PN . Outcome of autologous stem cell transplantation for AL amyloidosis in the UK. Br J Haematol. 2006; 134:417–25. 10.1111/j.1365-2141.2006.06204.x. 16822290

[27] Moreau P , Leblond V , Bourquelot P , Facon T , Huynh A , Caillot D , Hermine O , Attal M , Hamidou M , Nedellec G , Ferrant A , Audhuy B , Bataille R , et al. Prognostic factors for survival and response after high-dose therapy and autologous stem cell transplantation in systemic AL amyloidosis: a report on 21 patients. Br J Haematol. 1998; 101:766–69. 10.1046/j.1365-2141.1998.00772.x. 9674753

[28] Nader R , Zhen A , Angel-Korman A , Pavlovich SS , Pogrebinsky A , Doros G , Menn-Josephy H , Stern L , Sanchorawala V , Havasi A . Predictors and outcomes of acute kidney injury during autologous stem cell transplantation in AL amyloidosis. Nephrol Dial Transplant. 2022; 37:1281–88. 10.1093/ndt/gfab189. 34043009

[29] Manwani R , Cohen O , Sharpley F , Mahmood S , Sachchithanantham S , Foard D , Lachmann HJ , Quarta C , Fontana M , Gillmore JD , Whelan C , Hawkins PN , Wechalekar AD . A prospective observational study of 915 patients with systemic AL amyloidosis treated with upfront bortezomib. Blood. 2019; 134:2271–80. 10.1182/blood.2019000834. 31578202

[30] Kastritis E , Leleu X , Arnulf B , Zamagni E , Cibeira MT , Kwok F , Mollee P , Hájek R , Moreau P , Jaccard A , Schönland SO , Filshie R , Nicolas-Virelizier E , et al. Bortezomib, Melphalan, and Dexamethasone for Light-Chain Amyloidosis. J Clin Oncol. 2020; 38:3252–60. 10.1200/JCO.20.01285. 32730181

[31] Cai Y , Xu S , Li N , Li S , Xu G . Efficacy of Chemotherapies and Stem Cell Transplantation for Systemic AL Amyloidosis: A Network Meta-Analysis. Front Pharmacol. 2019; 10:1601. 10.3389/fphar.2019.01601. 32063846 PMC6997776

[32] Cibeira MT , Sanchorawala V , Seldin DC , Quillen K , Berk JL , Dember LM , Segal A , Ruberg F , Meier-Ewert H , Andrea NT , Sloan JM , Finn KT , Doros G , et al. Outcome of AL amyloidosis after high-dose melphalan and autologous stem cell transplantation: long-term results in a series of 421 patients. Blood. 2011; 118:4346–52. 10.1182/blood-2011-01-330738. 21828140 PMC3204906

[33] Palladini G , Milani P , Foli A , Obici L , Lavatelli F , Nuvolone M , Caccialanza R , Perlini S , Merlini G . Oral melphalan and dexamethasone grants extended survival with minimal toxicity in AL amyloidosis: long-term results of a risk-adapted approach. Haematologica. 2014; 99:743–50. 10.3324/haematol.2013.095463. 24213149 PMC3971085

[34] Sanchorawala V , Patel JM , Sloan JM , Shelton AC , Zeldis JB , Seldin DC . Melphalan, lenalidomide and dexamethasone for the treatment of immunoglobulin light chain amyloidosis: results of a phase II trial. Haematologica. 2013; 98:789–92. 10.3324/haematol.2012.075192. 23144200 PMC3640126

[35] Edwards CV , Rao N , Bhutani D , Mapara M , Radhakrishnan J , Shames S , Maurer MS , Leng S , Solomon A , Lentzsch S , Eisenberger A . Phase 1a/b study of monoclonal antibody CAEL-101 (11-1F4) in patients with AL amyloidosis. Blood. 2021; 138:2632–41. 10.1182/blood.2020009039. 34521113 PMC8703360

