# Addendum: Systemic AL amyloidosis: current approach and future direction

**DOI:** 10.18632/oncotarget.28478

**Published:** 2023-07-20

**Authors:** Maroun Bou Zerdan, Lewis Nasr, Farhan Khalid, Sabine Allam, Youssef Bouferraa, Saba Batool, Muhammad Tayyeb, Shubham Adroja, Mahinbanu Mammadii, Faiz Anwer, Shahzad Raza, Chakra P. Chaulagain

**Affiliations:** ^1^Department of Internal Medicine, SUNY Upstate Medical University, Syracuse, NY 13210, USA; ^2^University of Texas MD Anderson Cancer Center, Houston, TX 77030, USA; ^3^Department of Internal Medicine, Monmouth Medical Center, Long Branch, NJ 07740, USA; ^4^Department of Medicine and Medical Sciences, University of Balamand, Balamand, Lebanon; ^5^Department of Internal Medicine, Cleveland Clinic, Cleveland, OH 44195, USA; ^6^Department of Internal Medicine, UnityPoint Methodist, Peoria, IL 61636, USA; ^7^Department of Internal Medicine, Monmouth Medical Center, Long Branch, NJ 07740, USA; ^8^Hematology and Medical Oncology, Houston Methodist Cancer Center, Houston, TX 77094, USA; ^9^Department of Hematology-Oncology, Cleveland Clinic, Cleveland, OH 44195, USA; ^10^Department of Hematology-Oncology, Myeloma and Amyloidosis Program, Maroone Cancer Center, Cleveland Clinic Florida, Weston, FL 33331, USA

**This article has an addendum:** Research funding was obtained from the Cleveland Clinic Florida, Maroone Cancer Center, Malignant Hematology Research Fund.

Original article: Oncotarget. 2023; 14:384–394. 384-394. https://doi.org/10.18632/oncotarget.28415

